# Supplementary material for: Harnessing orbital Hall effect in spin-orbit torque MRAM
Source: Nat Commun. 2025 Jan 2;16:130. doi: 10.1038/s41467-024-55437-x (PMC11696791; doi:10.1038/s41467-024-55437-x)
Supplement: Supplementary file 1 — Supplementry Information [file 41467_2024_55437_MOESM1_ESM.pdf]

Harnessing Orbital Hall Effect in Spin-Orbit Torque MRAM

(Supplementary Materials)

Rahul Gupta,[1,*] Chloé Bouard,[2] Fabian Kammerbauer,[1] J. Omar Ledesma-Martin,[1] Arnab Bose,[1] Iryna Kononenko,[1] Sylvain Martin,[2] Perrine Usé,[2] Gerhard Jakob,[1,3] Marc Drouard,[2] and Mathias Kläui[1,3,4,†]

[1]*Institute of Physics, Johannes Gutenberg University Mainz, 55099 Mainz, Germany*

[2]*Antaios, 38240 Meylan, France*

[3]*Graduate School of Excellence Materials Science in Mainz, 55128, Mainz, Germany*

[4]*Department of Physics, Center for Quantum Spintronics, Norwegian University of Science and Technology, 7491 Trondheim, Norway*

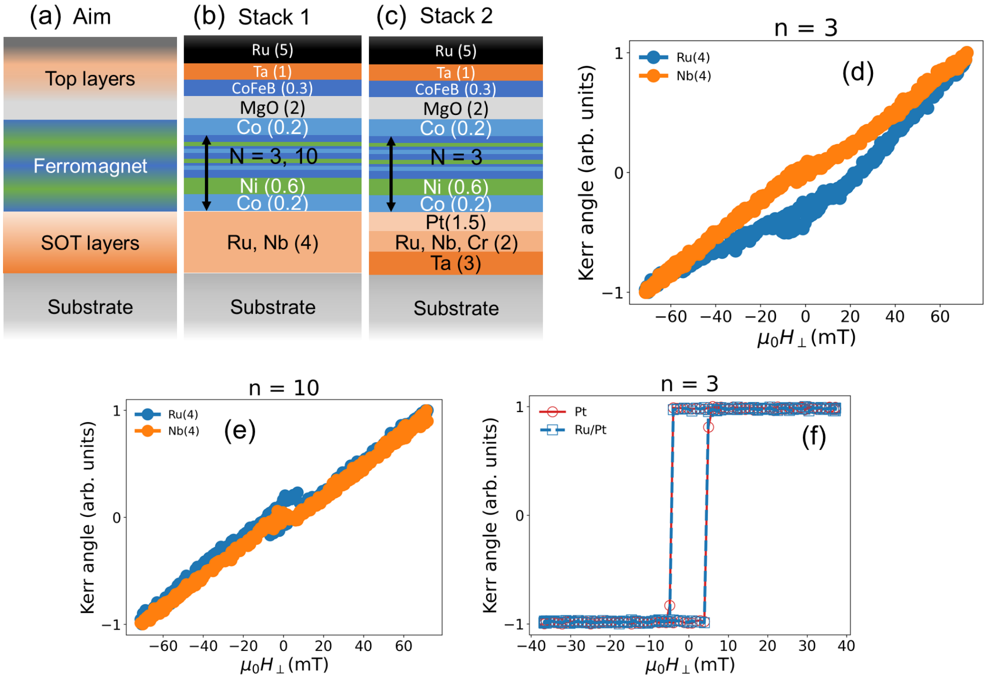

FIG. S1. (a-c) Cross-sectional schematic of different stacks utilized. Kerr signal versus out-of-plane applied field ($\mu_0 H_\perp$) for (d) stack 1 with n = 3, (e) stack 1 with n = 10, and (f) stack 2 with n = 3.

I. SAMPLE PREPARATION

Given the demands for high-density integration and efficient switching in SOT-MRAM systems, achieving perpendicular magnetic anisotropy (PMA) in ferromagnetic (FM) layers combined with various orbital Hall effect (OHE) materials is crucial. To attain PMA of the Co/Ni multilayer on different selected OHE layers, such as Ru, Nb, and Cr, different strategies were employed. The schematic of the stack is depicted in Fig. S1(a), comprising OHE-assisted SOT layers, Co/Ni multilayers serving as the free layer of SOT MRAM, an MgO barrier, and replicas of fixed layers of MTJs, such as MgO(2)/$Co_{20}Fe_{60}B_{20}$(0.3)/Ta(1)/Ru(5), here termed as top layers. Initially, the Co/Ni multilayers exhibit in-plane magnetic anisotropy when directly grown on Ru (4) and Nb (4), with similar results observed for different repetition numbers of $[Co/Ni]_n$ (n = 3 and 10) where n represents the repetition of Co/Ni multilayer. The complete stack is illustrated in Fig. 1(b), with MOKE results shown in Fig. S1(d,e).

Due to high spin-orbit coupling of Pt, it provides an advantage in our memory cell device by serving as an orbital-to-spin conversion layer. Hence, a Pt(1.5) layer

is inserted between $[Co/Ni]_3$ layers and OHE(2) layers, facilitating PMA and acting as an orbital to spin converter. The final stack, shown in Fig. S1(c), is verified by looking at the normalized Kerr signal as a function of out-of-plane applied magnetic field, as shown in Fig. S1(f). The stack exhibits PMA for all OHE layers and the coercivity of $[Co/Ni]_3$ on pure Pt and Ru/Pt samples is found to be the same ($\mu_0 H_c = 5$ mT). Subsequently, the same stack was deposited on a 100 mm Si/SiO_2 wafer for large-scale device fabrication, with PMA quality verified to be homogeneous across the entire wafer. Finally, a Pt(3.5) layer is deposited as the SOT layer instead of OHE(2)/Pt(1.5), serving as a reference sample to compare torques and switching current.

II. TORQUE MEASUREMENTS

S1. Torque efficiency without correction factor

To extract torque efficiencies, we employed standard harmonic Hall measurements in the presence of applied magnetic fields at room temperature (as detailed in the methods section of the main manuscript). Specifically, we measured the first harmonic Hall voltage as a function of in-plane applied fields, denoted as $\mu_0 H_x$ and $\mu_0 H_y$. Subsequently, utilizing Eq. 2 mentioned in the main text, we extract the DL and FL fields. Following this procedure, we defined the efficiencies of DL (ξ^E_{DL}) and FL (ξ^E_{FL}) torques per unit electric field as follows[1],

$$\xi^E_{DL(FL)} = \frac{2e}{\hbar}\frac{\mu_0 M_s t_{FM}}{E} H_{DL,y(FL,x)}, \tag{S1}$$

where e, $\hbar$, $\mu_0 M_s$, and t_{FM} represent the electronic charge, the reduced Planck's constant, the saturation magnetization of the FM layer, and the thickness of the FM layer, respectively. Figure S2(a) and S2(b) show the DL(FL) torque efficiencies per unit electric field ($\xi^E_{DL(FL)}$) and per unit current density ($\xi^J_{DL(FL)}$) for all OHE-assisted SOT lines, respectively. Notably, the FL torque efficiencies are approximately 10% of the DL torque efficiencies across all samples. This observation suggests that the switching mechanism discussed in the main text is predominantly influenced by the DL torque.

Furthermore, we observe that the initially measured torque efficiencies for the Cr OHE layer exhibit the highest magnitude compared to other OHE layers utilized in this study. This finding contrasts with the theoretically predicted orbital Hall conductivity (OHC) of Ru, Nb, and Cr, as Ru exhibits a larger OHC compared to Cr[2]. Previous research has demonstrated that the magnitude of torque efficiency is susceptible to artifact correction, such as the anomalous Hall effect (AHE) and planar Hall effect (PHE), as discussed in Ref.[3]. Therefore, we proceed to measure the PHE and AHE for all samples, as elaborated in the next section.

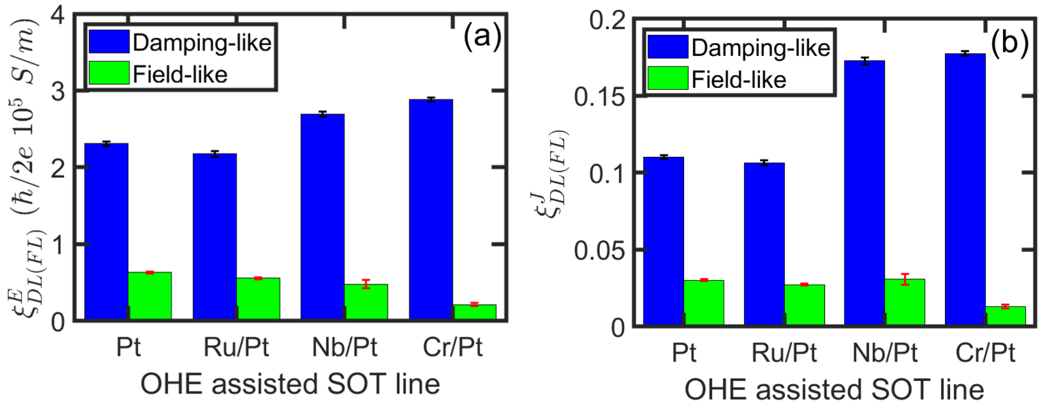

FIG. S2. (a) Torque efficiency per unit electric field ($\xi^E_{DL(FL)}$) and (b) per unit current density ($\xi^J_{DL(FL)}$) for different OHE-assisted SOT lines.

S2. Anomalous Hall effect

The AHE voltages (V_{AHE}) are measured for all samples in the presence of an out-of-plane applied magnetic field ($\mu_0 H_z$). V_{AHE} is determined by extrapolating from positive and negative high fields to zero field, as illustrated by the dotted line in Fig. S3(a). Figures S3(a-c) show the AHE voltage for all OHE-assisted SOT line samples. The difference between the two intercepts, such as positive and negative, at $\mu_0 H_z = 0$ corresponds to $2V_{AHE}$.

S3. Planar Hall effect

The PHE voltages, denoted as $V_{PHE}(\phi)$, were measured for all samples in the presence of an in-plane applied magnetic field ($\mu_0 H_{applied}$). The PHE voltage exhibits $\sin(2\phi)$ dependence, where ϕ represents the angle between the current and the $\mu_0 H_{applied}$ in the film plane[4]. A typical behavior of V_{PHE} as a function of ϕ is depicted in Fig. S3(d) at an applied field of 800 mT for the Ru(2)/Pt(1.5) sample. V_{PHE} is defined as half of the peak-to-peak voltage and extracted by fitting the experimental data with $V_{PHE}\sin(2\phi)$. Figures S4(a-d) represent V_{PHE} as a function of $\mu_0 H_{applied}$ for all samples.

The parameter η is defined as the ratio of the resistance due to the PHE at saturation of $\mu_0 H_{applied}$ to that of the AHE. It is noteworthy that, in our case, the significance of the PHE relative to the AHE is introduced as a correction factor in the analysis, which is discussed in the following section.

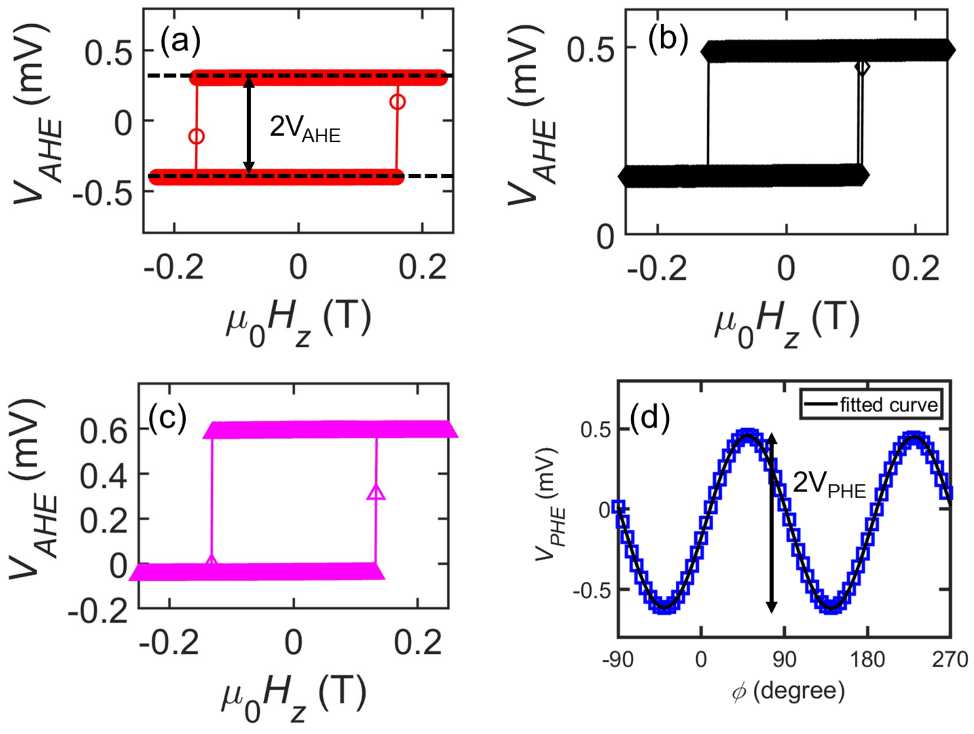

FIG. S3. Anomalous Hall voltage as a function of $\mu_0 H_z$ for (a) Pt(3.5), (b) Nb(2)/Pt(1.5), and (c) Cr(2)/Pt(1.5). (d) Planar Hall voltage as a function of in-plane angle (ϕ) at 800 mT applied field.

S4. Torque efficiencies with and without correction factor

The corrected DL torque efficiency per unit electric field (ξ^E_{DL}) and per unit current density (ξ^J_{DL}) are defined by Eq. 1 in the main manuscript. The ξ^E_{DL} and ξ^J_{DL} with and without correction factor are shown in Fig. S5(a,b), respectively. Note that the DL torque efficiency is found to be larger for the Ru(2)/Pt(1.5) sample than for pure Pt(3.5). Since the sign of the torque efficiency is positive in all samples, it indicates that the dominant mechanism is the orbital Hall effect. Specifically, Ru, Nb, and Cr exhibit a positive sign of OHC, while Nb and Cr exhibit a negative sign of the spin Hall conductivity[2,5].

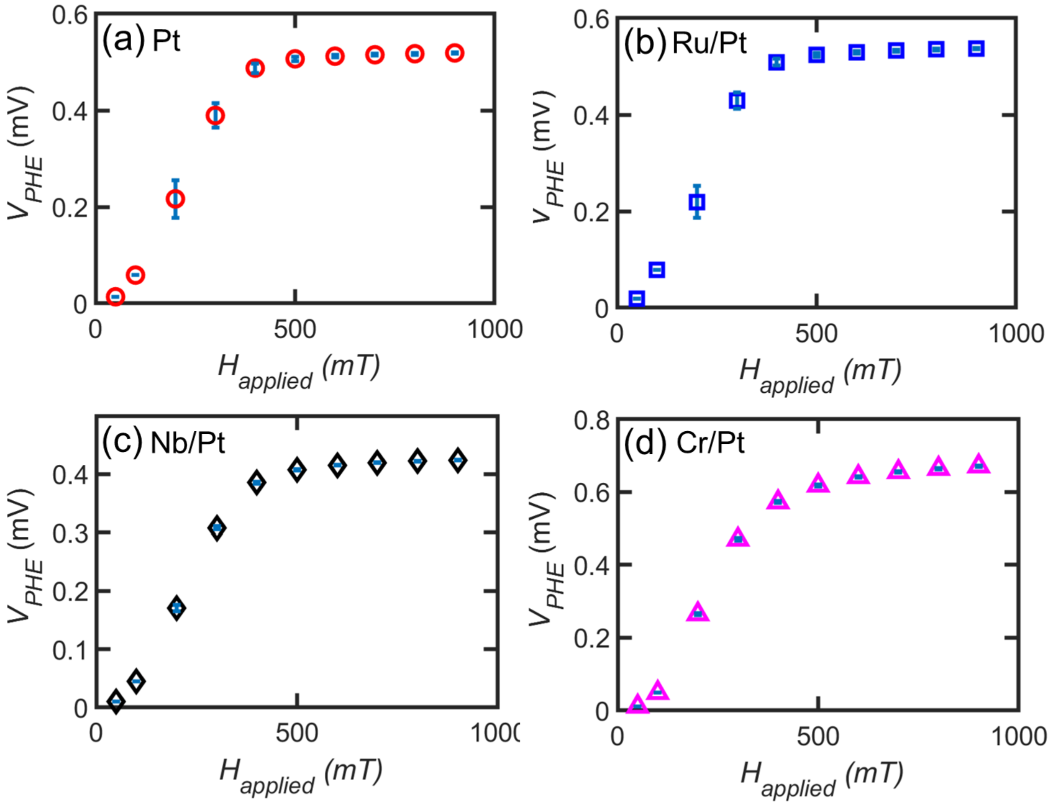

FIG. S4. Planar Hall voltage as a function of in-plane applied field for (a) Pt(3.5), (b) Ru(2)/Pt(1.5), (c) Nb(2)/Pt(1.5), and (d) Cr(2)/Pt(1.5).

III. MAGNETIZATION MEASUREMENTS

A Quantum Design Superconducting Quantum Interference Device (SQUID) magnetometer was employed to measure the magnetic moments of the samples by applying an in-plane magnetic field. To quantify the magnetization, the measured moment values were normalized to the magnetic volume. This volume was determined by taking into account the film thickness—which was set by adjusting the deposition rate—and the precise area of the sample, which was measured using optical microscopy. The magnetization of all the samples is shown in Fig. S6.

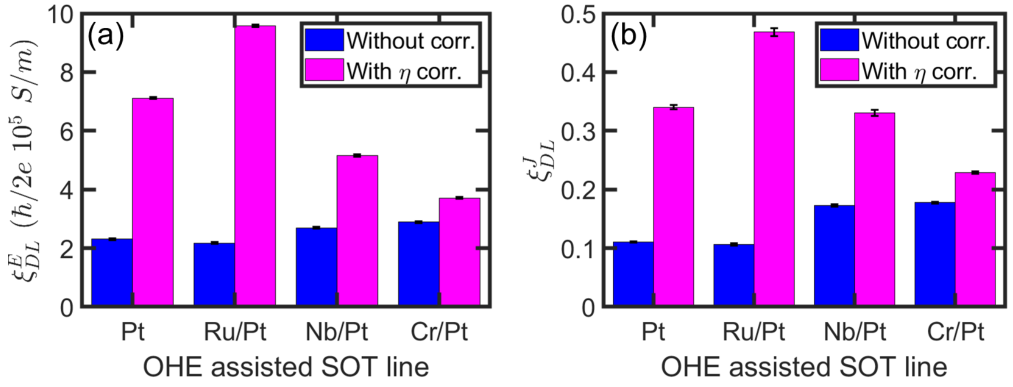

FIG. S5. (a) Damping-like torque efficiency per unit electric field (ξ^E_{DL}) and (b) per unit current density (ξ^J_{DL}) with and with η correction for different OHE-assisted SOT lines.

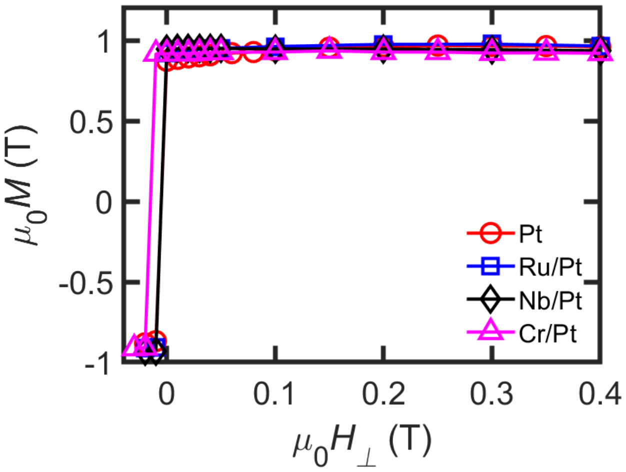

FIG. S6. Magnetization vs. $\mu_0 H_z$ for all Pt(3.5), Ru(2)/Pt(1.5), Nb(2)/Pt(1.5), and Cr(2)/Pt(1.5). The full stack can be seen in Fig. S1c.

IV. CURRENT INDUCED MAGNETIZATION SWITCHING

S1. Switching current density: Effect of pulse duration for >250 devices

To utilize the enhanced torques in the current-induced switching experiment, we measured the switching current densities in more than 250 devices for different pulse durations at 50 mT in-plane applied field along the x-direction. Fig. S7(a,b) show the critical current densities as a function of device resistance for all OHE-

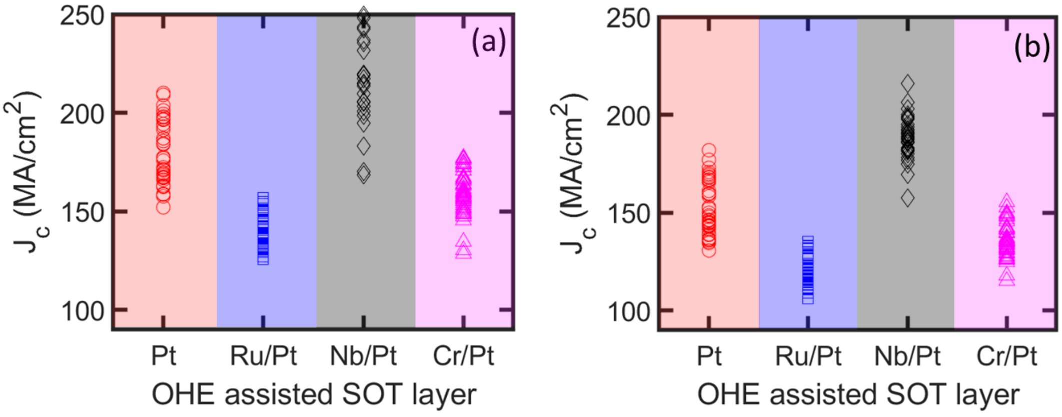

FIG. S7. Critical current density (J_c) for different OHE assisted SOT line (R) at $\mu_0 H_x$ = 50 mT for (a) 3 ns, (b) 5 ns pulse duration.

assisted SOT lines for 3 ns and 5 ns pulse durations, respectively. Here, we found that the average critical current density is 20% smaller in the Ru(2)/Pt(1.5) SOT line compared to Pt(3.5), which is consistent with the enhancement in the orbital torques.

S2. Thermal stability and anisotropic fields in >250 devices

One of the most important features of an MRAM device is the time that the information will be reliably stored. This is characterized by the data retention time τ, that depends on the thermal stability factor Δ via the Arrhenius law:

$$\tau = \tau_0 e^{\Delta} \tag{S2}$$

where τ_0 (1 ns) is the inverse of the attempt frequency. Δ is defined in terms of the energy barrier E_B between the parallel (P) and antiparallel (AP) states of the MTJ, I the applied current, H the magnetic field, I_c the extrapolated switching current at τ_0, H_k the magnetic anisotropy field, k_B the Boltzmann constant and T the temperature. E_B can be defined as:

$$E_B = \frac{H_k M_s V}{2} \tag{S3}$$

where H_k is the effective perpendicular magnetic anisotropy field, M_s the saturation magnetization and, V the volume of the free layer. In order to characterize the stability factor Δ and, in consequence, the data retention time τ, a magnetic field is swept constantly several times (50 to 300 in this study) and extracting

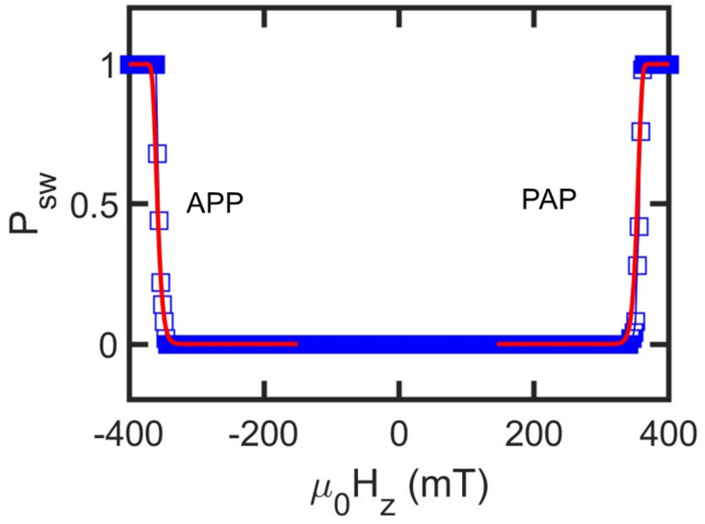

FIG. S8. Probability of switching $P_{\rm sw}$ of Ru(2)/Pt(1.5) device as a function of $\mu_0 H_z$ for anti-parallel to parallel (APP) and parallel to anti-parallel (PAP) states. The parameters used during the measurements are: field sweep rate (r) 118.8 mT/s, 50 cycles, H_{off} = 1.2 mT.

Δ from its distribution. The measured switching probability P_{SW} is linked to Δ by[6,7]:

$$P_{SW} = 1 - exp\left(- \frac{H_k f_0 \sqrt{\pi}}{2r\sqrt{\Delta}} erfc\left(\sqrt{\Delta}\left(1 - \frac{|H - H_{off}|}{H_k}\right)\right)\right) \tag{S4}$$

where H_k is the anisotropy field, H_{off} is the offset field, f_0 is the attempt frequency (1 GHz), and r is the field sweep rate. P_{SW} is calculated by measuring the number of times that a device switches at different points in the field sweeps, and then Δ and H_k are fitted for both AP to P and P to AP. A typical behavior of $P_{\rm sw}$ as a function of the applied field is shown in Fig. S8. The H_k and Δ, and their average values, are shown in Fig. S9-S11 for 3, 5, and 10 ns pulse durations, respectively. Note that the average H_k and Δ from more than 250 devices for all OHE-assisted SOT samples are found to be constant within the error bars.

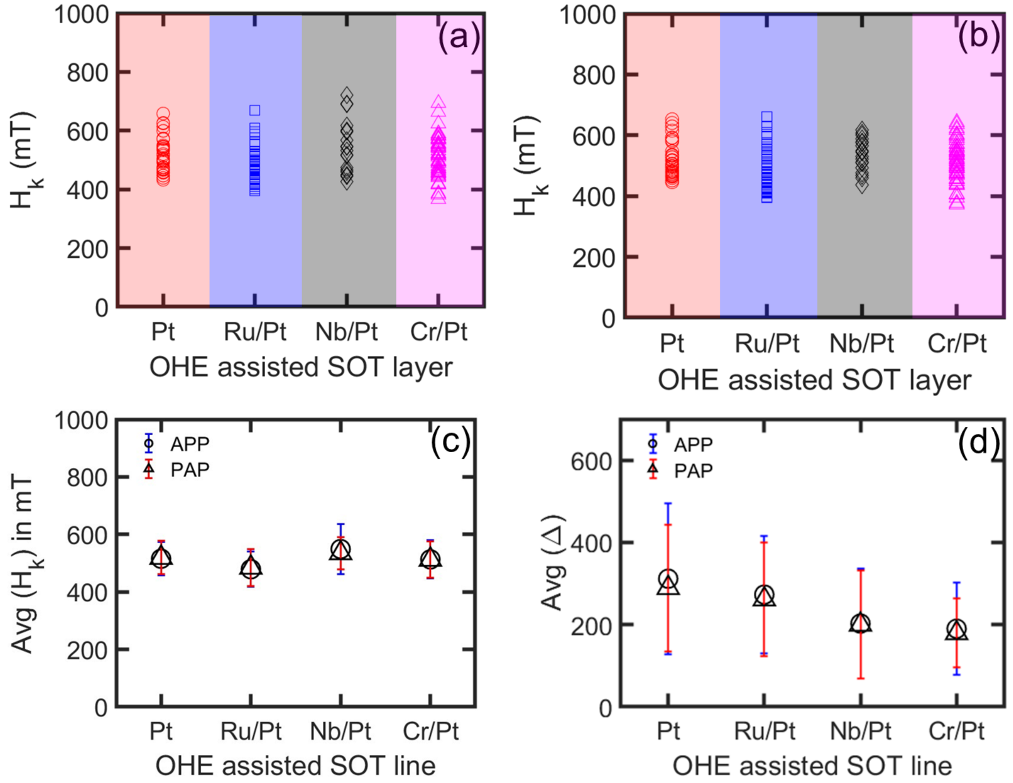

FIG. S9. Anisotropic field (H_k) for different OHE assisted SOT line for (a) anti-parallel to parallel (APP) state, and (b) parallel to anti-parallel (PAP) states. (c) Average of H_k for both APP and PAP states, and (d) average thermal stability (Δ) for both APP and PAP states. These plots correspond to a 3 ns pulse duration.

OHE layers	$\mu_0 M_s$ (mT)	$\mu_0 H_c$ (mT)	$\mu_0 H_k$ (mT)	ξ^J_{DL}	Avg(J_c) (MA/cm^2)	Avg(Δ)
Pt(3.5)	965.2±40	5.0±0.1	518.1±58.7	0.33±0.003	129.58±10.88	286±165
Ru(2)/Pt(1.5)	976.5±35	4.9±0.1	482.6±56.8	0.46±0.006	99.04±5.99	271±149
Nb(2)/Pt(1.5)	941.8±44	9.0±0.1	503.4±93.2	0.33±0.005	159.19±13	257±149
Cr(2)/Pt(1.5)	927±38	10±0.1	513.2±62.7	0.22±0.002	113±7.4	190±108

TABLE ST1. Comparison of different parameters for all stacks used. Avg(J_c) and Avg(Δ) correspond to a 10 ps pulse duration. The parameters in the first two columns correspond to thin film stacks, while the remaining parameters are obtained from the device.

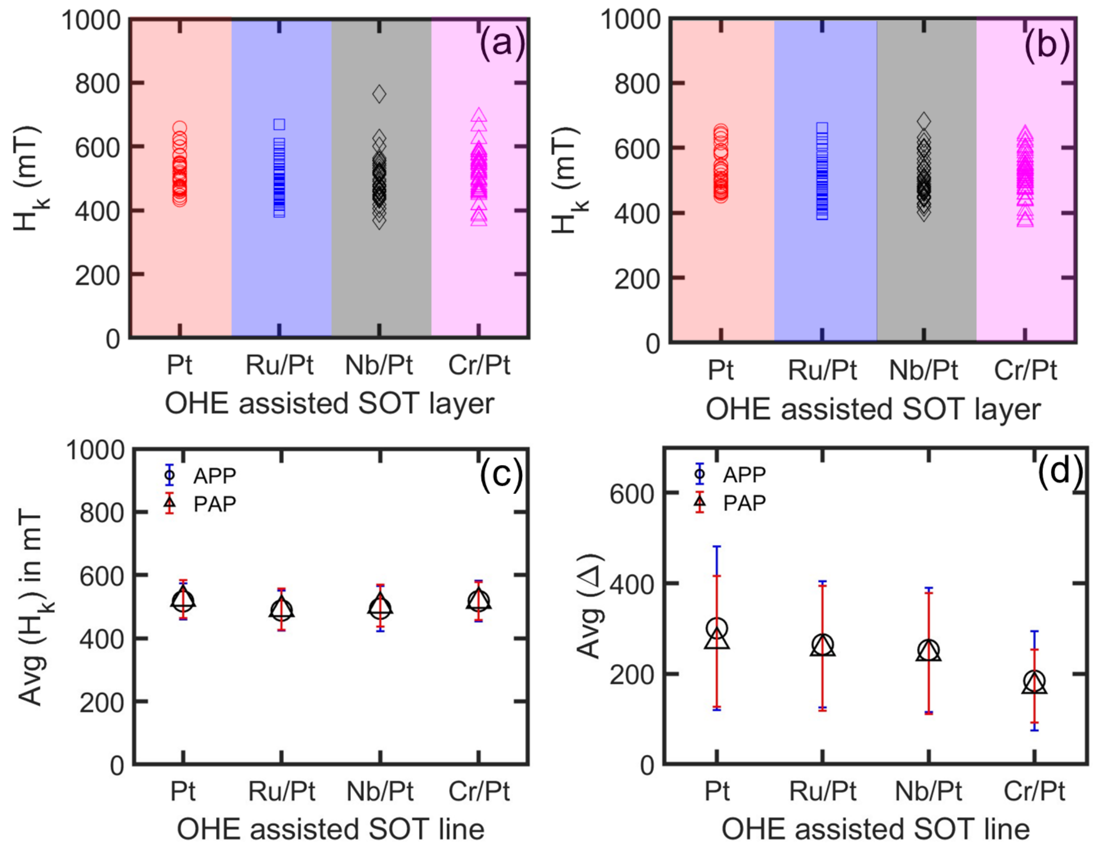

FIG. S10. Anisotropic field (H_k) for different OHE assisted SOT line for (a) anti-parallel to parallel (APP) state, and (b) parallel to anti-parallel (PAP) states. (c) Average of H_k for both APP and PAP states, and (d) average thermal stability (Δ) for both APP and PAP states. These plots correspond to a 5 ns pulse duration.

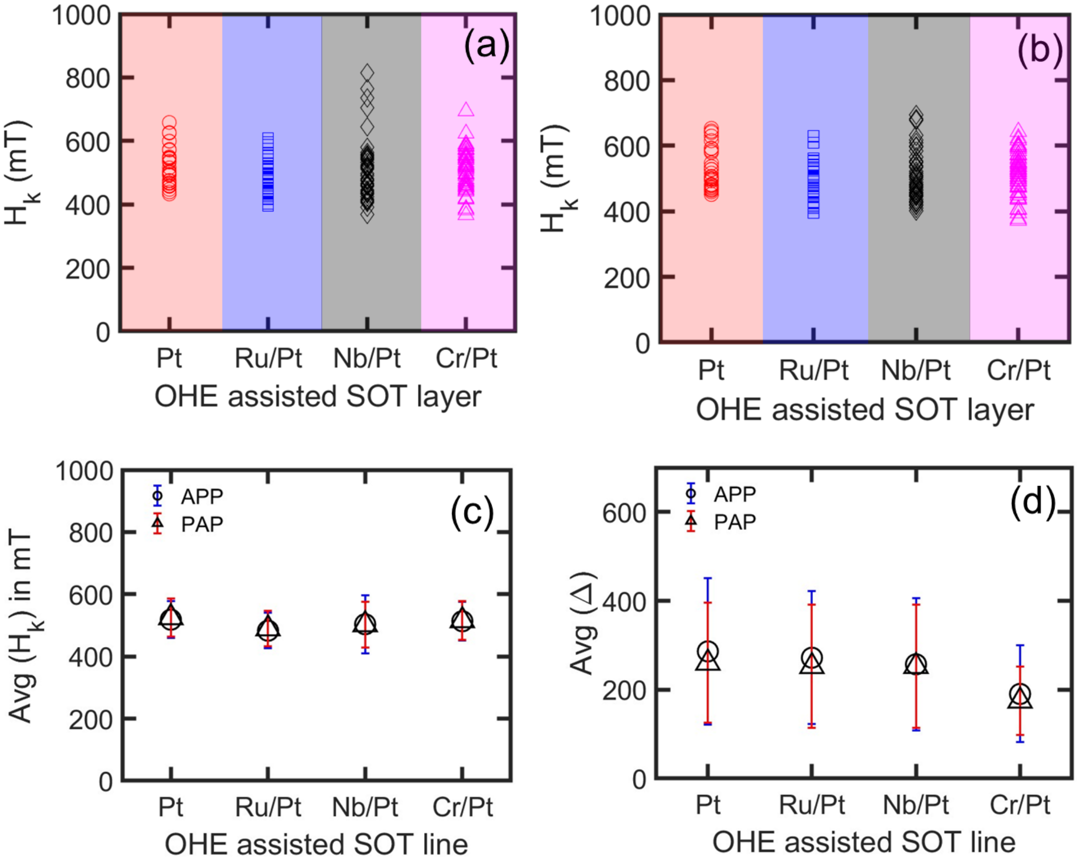

FIG. S11. Anisotropic field (H_k) for different OHE assisted SOT line for (a) anti-parallel to parallel (APP) state, and (b) parallel to anti-parallel (PAP) states. (c) Average of H_k for both APP and PAP states, and (d) average thermal stability (Δ) for both APP and PAP states. These plots correspond to a 10 ns pulse duration.

* rahul.gupta.phy@outlook.com
† klaeui@uni-mainz.de
[1] M. Hayashi, J. Kim, M. Yamanouchi, and H. Ohno, Phys. Rev. B **89**, 144425 (2014).
[2] L. Salemi and P. M. Oppeneer, Phys. Rev. Mater. **6**, 095001 (2022).
[3] S. Karimeddiny, J. A. Mittelstaedt, R. A. Buhrman, and D. C. Ralph, Phys. Rev. Appl. **14**, 024024 (2020).
[4] N. Roschewsky, E. S. Walker, P. Gowtham, S. Muschinske, F. Hellman, S. R. Bank, and S. Salahuddin, Phys. Rev. B **99**, 195103 (2019).
[5] D. Go, H.-W. Lee, P. M. Oppeneer, S. Blügel, and Y. Mokrousov, Phys. Rev. B **109**, 174435 (2024).
[6] L. Thomas, G. Jan, J. Zhu, H. Liu, Y.-J. Lee, S. Le, R.-Y. Tong, K. Pi, Y.-J. Wang, D. Shen, *et al.*, J. Appl. Phys. **115** (2014), 10.1063/1.4870917.
[7] Y. Wu, K. Garello, W. Kim, M. Gupta, M. Perumkunnil, V. Kateel, S. Couet, R. Carpenter, S. Rao, S. Van Beek, K. Vudya Sethu, F. Yasin, D. Crotti, and G. Kar, Phys. Rev. Appl. **15**, 064015 (2021).
