# Supplementary material for: Harnessing orbital Hall effect in spin-orbit torque MRAM
Source: Nat Commun. 2025 Jan 2;16:130. doi: 10.1038/s41467-024-55437-x (PMC11696791; doi:10.1038/s41467-024-55437-x)
Supplement: Supplementary file 2 — Transparent Peer Review file [file 41467_2024_55437_MOESM2_ESM.pdf]

nature portfolio

Peer Review File

Harnessing Orbital Hall Effect in Spin-Orbit Torque MRAM

Corresponding Author: Professor Mathias Kläui

This manuscript has been previously reviewed at another journal. This document only contains information relating to versions considered at Nature Communications.

This file contains all reviewer reports in order by version, followed by all author rebuttals in order by version.

Version 0:

Reviewer comments:

Reviewer #1

(Remarks to the Author)
I appreciate the authors effort to address the comments of my previous report in another journal. they succesfully added the relevant sample details as supplementary material) which I believe indeed support the claims so far.

the manuscript can be published in its present form.

Reviewer #2

(Remarks to the Author)
The authors have addressed all issues raised before.

Open Access This Peer Review File is licensed under a Creative Commons Attribution 4.0 International License, which

permits use, sharing, adaptation, distribution and reproduction in any medium or format, as long as you give appropriate credit to the original author(s) and the source, provide a link to the Creative Commons license, and indicate if changes were made.
In cases where reviewers are anonymous, credit should be given to 'Anonymous Referee' and the source.
The images or other third party material in this Peer Review File are included in the article’s Creative Commons license, unless indicated otherwise in a credit line to the material. If material is not included in the article’s Creative Commons license and your intended use is not permitted by statutory regulation or exceeds the permitted use, you will need to obtain permission directly from the copyright holder.
To view a copy of this license, visit https://creativecommons.org/licenses/by/4.0/
